# Comparability of estimates and trends in adolescent sexual and contraceptive behaviors from two national surveys: National Survey of Family Growth and the Youth Risk Behavior Survey

**DOI:** 10.1371/journal.pone.0253262

**Published:** 2021-07-30

**Authors:** Laura D. Lindberg, Rachel H. Scott, Sheila Desai, Zoe H. Pleasure

**Affiliations:** 1 Guttmacher Institute, New York, New York, United States of America; 2 London School of Hygiene and Tropical Medicine, London, United Kingdom; Montclair State University, UNITED STATES

## Abstract

**Objective:**

To compare adolescents’ reports of sexual and contraceptive behaviors between the National Survey of Family Growth (NSFG) and the Youth Risk Behavior Survey (YRBS).

**Methods:**

For each survey, we estimated the year- and sex-specific prevalence of sexual and contraceptive behaviors among a comparably defined sample of US respondents ages 15–19 currently attending high school. We used logistic regression to test for changes in prevalence from 2007–2019 and conducted sensitivity analyses to investigate between-survey differences.

**Results:**

We found differences in both prevalence and trends between the YRBS and NSFG when limited to a comparably defined sample. Compared to the NSFG, adolescents in the YRBS were more likely to report being sexually experienced, less likely to report use of prescription methods for both sexes, and less likely to report condoms among males. Only the YRBS estimated significant declines in sexual experience for both sexes, and significant increases in prescription methods and declines in condom use among males. Differences between surveys in the prevalence of specific contraceptive methods reflected greater combined use of methods in the NSFG. We identified differences in question-wording and other aspects that may influence these differential patterns.

**Conclusions:**

The NSFG and YRBS produced inconsistent prevalence estimates and trends for sexual and contraceptive behaviors among in-school adolescents. Further efforts to improve these national surveillance systems are critical to inform policy and research efforts that support adolescent sexual and reproductive health and wellbeing.

## Introduction

Sexual development is a critical developmental task of adolescence that can be promoted and supported by public health policy. Trends and differentials in adolescent sexual behaviors are used to identify the need for and monitor the progress of health promotion activities [1–3]. Currently, two federal surveys, the National Survey of Family Growth (NSFG) and the Youth Risk Behavior Survey (YRBS), provide ongoing and comprehensive surveillance of national trends in adolescent sexual behavior [4, 5]. Other nationally representative survey systems, such as the National Longitudinal Survey of Adolescent and Adult Health (Add Health), the National Longitudinal Survey of Youth (NLSY), the National Health and Nutrition Examination Survey (NHANES), and the Behavioral Risk Factor Surveillance System (BRFSS), have also been used to study and monitor adolescent sexual health. However, these surveys are limited by nonrecent data on adolescent behaviors (Add Health and NLSY), few adolescent health measures and small sample sizes of adolescents (NHANES), and the exclusion of respondents younger than age 18 (BRFSS). Thus, the NSFG and YRBS serve as critical resources for adolescent sexual health research. Understanding the differences in findings between the NSFG and the YRBS and key drivers of these differences are essential to develop public health priorities and objectives, including the monitoring of adolescent sexual behavior in the Surgeon General’s new Healthy People 2030 program [2].

Prior studies that directly compare estimates for adolescent sexual behaviors across multiple United States’ (U.S.) surveys have found significant differences in levels and trends after adjusting for variations in sample compositions [6, 7]. Comparing the 1995 NSFG and YRBS for females, Santelli et al. found significant differentials in estimates of sexual behaviors after limiting each survey to a comparable subsample of high school students [6]. For example, the estimates of sexual experience differed by sixteen percentage points. Although the 1995 NSFG did not include males, comparisons between the male respondents in the 1995 YRBS, the 1995 National Longitudinal Survey of Adolescent Health (Add Health) and the 1995 National Survey of Adolescent Males (NSAM) found that the YRBS estimated higher rates of sexual experience than the other surveys. There were also differences in 1995 contraceptive use estimates between surveys. However, time trends were generally comparable between surveys. Although males were included in the NSFG starting in 2002, more recent direct comparisons of sexual behaviors and contraceptive use between the NSFG and YRBS are lacking, and the generalizability of prior findings to more recent data is unknown.

Changes during the intervening two decades suggest that more recent data collection has occurred in a different context, which may influence reporting patterns. Relevant attitudinal shifts include less conservative social norms around adolescent sexual behavior generally, as well as increased acceptance of same-sex behaviors [8, 9]. In more recent years, expansion of contraceptive coverage in health insurance has expanded access to a breadth of contraceptive methods and guidelines supporting the use of LARC for adolescents have increased uptake of these methods [10]. Additionally, with high school dropout rates declining by more than half from 1995 to 2017, the school-based YRBS may have become more comparable to the household-based NSFG over time, potentially reducing previously observed differences [11]. At the same time, recent data collection efforts have faced broadly declining response rates and decreasing trust in the survey experience, which may influence respondents’ reporting of sensitive sexual behaviors [12, 13]. Together, these changes highlight the need for reevaluating patterns of reporting between the NSFG and YRBS.

Differences between NSFG and YRBS estimates and trends may reflect differences in the surveys’ designs, including sampling frame or measurement issues. The YRBS is a nationally representative sample of high school students limited to studying health behaviors. In contrast, the NSFG is a representative household sample addressing topics for the full reproductive age range that includes adolescents in and out of school. The exclusion of non-students from the YRBS sample likely influences the observed estimates, as out-of-school adolescents are more likely to engage in sexual activity and other health risk behaviors [14]. However, differences in observed estimates between the two surveys may remain even after harmonizing the samples [6, 7], suggesting other measurement issues such as mode of administration or question wording.

Because NSFG and YRBS data are widely used to inform and monitor research, policy, and programs addressing adolescent sexual and reproductive health, it is important to consider issues of comparability in both trends and levels of adolescent sexual behavior between these surveys. This analysis compares adolescents’ reports of their sexual and contraceptive behaviors over the period from 2007–2019 in the YRBS and NSFG, restricting each sample to a mutually comparable population. We examine whether analyses of each survey system independently reach similar conclusions about trends and levels of these behaviors. Understanding how findings differ between these two national survey systems provides insight into measurement issues that impact the surveillance of adolescent sexual behavior. Given the federal effort to collect data under these two survey systems, their primacy in research and public health surveillance, and no clear existing guidance on the relative usefulness of each survey to study adolescent sexual health, this research will provide needed information to improve public health data collection and monitoring.

## Methods

### Data

The NSFG is a nationally representative survey of the noninstitutionalized population of reproductive-aged women and men (ages 15–49) in the U.S., conducted in-person in households by the National Center for Health Statistics (NCHS). Neither the YRBS nor NSFG ask directly about gender identity and some respondents may identify as different from their biological sex at birth. The survey collects detailed information on fertility-related behaviors, including sexual activity and contraceptive use. We use data from interviews conducted continuously from June 2006-June 2010 and September 2011-September 2019 [5, 15–18]. The NSFG uses both computer-assisted personal interviewing (CAPI), and audio computer-assisted self-interviews (ACASI).

The YRBS is a nationally representative school-based survey of high school students conducted biennially by the Centers for Disease Control and Prevention (CDC), designed to measure health behaviors [19]. Paper and pencil classroom surveys are administered to public and private high school students. We limit our analysis to the period 2007–2019.

Each survey used multistage stratified clustered sampling and oversampled Black and Hispanic respondents; the NSFG also oversampled adolescents. NSFG sampling weights are provided for each two-year data file listed in Table 1 and are provided for each of the two-year period within the 2006–2010 data file. The result is six nationally representative and non-overlapping two-year periods, which we refer to by their midpoints (2007, 2009, 2012, 2014, 2016 and 2018). Weights for the biennial data from the YRBS produce nationally representative samples of high school students for each survey year (2007, 2009, 2011, 2013, 2015, 2017 and 2019). Table 1 provides a summary of the purpose and design of each survey, with additional information and publicly available datasets provided online: NSFG (www.cdc.gov/nchs/nsfg/); YRBS (www.cdc.gov/healthyyouth/data/yrbs/). Methods of data collection and dissemination of the public-use datasets were approved by the NCHS Institutional Review Board’s protections of human subjects.

**Table 1:** Description of survey design and implementation of Youth Risk Behavior Survey (YRBS) and National Survey of Family Growth (NSFG).

	YRBS	NSFG
**Purpose**	Health risk behaviors	Fertility, family, health
**Survey design**	Cross-sectional	Cross-sectional
**When fielded**	Biennially	Continuous data collection released in two-year datasets
**Survey sample**	Nationally representative sample of high school students in grades 9–12	Nationally representative household survey of individuals aged 15-44/49*
**Sample used in analysis**	15–18+-year-olds, grades 9–12	In-school, 15-19-year-olds
**Survey rounds used in analysis**	2007, 2009, 2011, 2013, 2015, 2017, 2019	2006–2010, 2011–2013, 2013–2015, 2015–2017, 2017–2019
**Sampling**	Multistage, stratified, clustered	Multistage, stratified, clustered
**Oversampled**	Black & Hispanic students	Non-Hispanic Black respondents, Hispanic respondents and teens aged 15–19
**Sampling frame**	Public, Catholic and other private schools	Households
**Survey mode**	Paper & pencil interview, self-administered	Face-to-face, administered by interviewer; Audio Computer-Assisted Self-Interviewing (ACASI)
**Interview location**	School	Home
**Response rate**	60–71%	63–77%
**Length**	1 class period, 45 minutes	Average of 47 minutes for 15-19-year-olds
**Parental permission**	Active or passive depending on school	Active for minors
**Survey agency**	Centers for Disease Control, Division of Adolescent and School Health	Centers for Disease Control, National Center for Health Statistics

* 2015–17, 2017–19 extended sample to age 49.

### Analytic sample

To create comparable analytic samples, we restricted both surveys to respondents that met common age and school status criteria. We limited the original YRBS sample to include only 15–18+ year-olds in grades 9–12. The YRBS analytic sample included 91,392 respondents from 2007–2019, or 89% of the original YRBS sample; all excluded respondents who were under 15 years of age. Because the YRBS identified respondents as age 18 or older, it was impossible to distinguish the single year of age in this category. In contrast, the NSFG identified single year of age. We estimated that only 3% of NSFG respondents attending high school were older than age 19, so we restricted the original NSFG sample to respondents ages 15–19 to be most aligned with the YRBS. Among these respondents, we further limited the NSFG sample to those who were currently in grades 9–12, attended grades 9–12 in the last 30 days, or, if interviewed in the summer, attended school in May or any subsequent months in grades 9–11. We excluded NSFG respondents who reported having completed grade 12 or who had received a high school degree or general equivalency degree. The final NSFG analytic sample from 2007–2019 included 8,106 respondents, or 64% of the original 15-19-year-old NSFG sample.

### Variables

We created dichotomous indicators of sexual behaviors and contraceptive use at last sex. The question items varied slightly across surveys (see S1 Table). Sexual behaviors examined included ever had sex and currently sexually active. To measure ever had sex, the YRBS asks all respondents: "*Have you ever had sexual intercourse*?" and does not provide an explicit definition of intercourse. The NSFG asks: "*At any time in your life*, *have you ever had sexual intercourse with a woman/man*, *that is*, *made love*, *had sex*, *or gone all the way*?" NSFG respondents were explicitly told by the interviewer not to count oral sex, anal sex, heavy petting, or other forms of sexual activity that do not involve vaginal penetration, or sex with a same-sex partner [20]. Through follow-up questions, the surveys then identify respondents who had sexual intercourse in the last three months; the YRBS asks a direct question about partners during the past three months, while the NSFG’s measure is based on the date of last sex with the last sexual partner.

In addition to the sexual behavior variables, we also examined measures of contraceptive use at last sex. The YRBS collects this contraceptive information using two questions. The first asks respondents if they or their partner used a condom the last time they had sexual intercourse. The second question asks: "*The last time you had sexual intercourse*, *what one method did you or your partner use to prevent pregnancy*?" and respondents are instructed to select one response. We combined responses from both questions.

In contrast, the NSFG asks about contraceptive method used at last sex with the last partner, limited to the last three months, and allows respondents to report multiple methods. For comparability between surveys, we create measures of any contraceptive method use, prescription method use (IUD, implant, shot, pill, patch, and ring), condom use (alone or in combination with prescription methods or withdrawal), and withdrawal use or other methods (alone or in combination with condom use; referred to here as "withdrawal" for brevity). In both surveys, all behaviors were asked of both female and male respondents, so the contraceptive use reported may refer to their partner’s use (e.g., condom use for females).

### Analysis

All analyses are conducted with the analytic sample. Within each survey, we calculate the prevalence of each outcome and the 95% confidence interval (CI) for each survey year by sex. We identified non-overlapping confidence intervals for point estimates between paired or adjacent years within each survey [21]. Next, within each survey, we estimate separate logistic regression models by sex with each outcome as the dependent variable and survey year as the independent variable to identify significant changes in prevalence estimates since 2007. We describe trends in each survey and highlight differences in patterns of reporting between the NSFG and YRBS.

We also conduct a range of sensitivity analyses. First, to assess how dual method use reporting contributes to the differences in prevalence estimates by survey, we pool the data across survey years to analyze the 2007–2019 prevalence of single or dual use of methods. Second, we test interactions between survey and age to test if differences between survey systems were consistent by age. Third, to test the influence of differences in interview mode, we compare the YRBS self-administered question of ever had sex with the NSFG ACASI measure (there is no ACASI measure for contraceptive use to compare). Fourth, we consider if the YRBS respondents could be reporting same-sex behaviors instead of penile-vaginal sex. Finally, we compare estimates of respondents’ recent alcohol use and self-reported height and weight, three non-sexual measures assessed in both surveys, to identify if patterns of differences between surveys parallel those observed for sexual and contraceptive behaviors.

All analyses use sampling weights provided for each survey with the *svy* command prefix in Stata 16.1 to adjust for the unique complex survey design of the specific dataset [19].

## Results

Overall, the analytic samples in the analysis include 91,392 YRBS respondents and 8,106 NSFG respondents. In each year, the YRBS analytic sample has between about 12,000–15,000 respondents overall, more than 4,000 of whom who have ever had sex, and more than 3,000 who are current sexually active. In contrast, in each period the NSFG analytic sample has between about 1,200 and 1,600 respondents, with fewer than 600 respondents sexually experienced or sexually active. Within the analytic samples, respondents in both surveys had generally similar demographic distributions by sex, race/ethnicity, and age. Both surveys showed an increase over time in the proportion identified as Hispanic (Table 2).

**Table 2:** Characteristics of YRBS and NSFG analytic* samples, by survey year.

	YRBS								NSFG†						
	2007	2009	2011	2013	2015	2017	2019	Total	2007	2009	2012	2014	2016	2018	Total
**Number of respondents**	** **							** **							** **
Total	*12589*	*14661*	*13734*	*12094*	*13814*	*12681*	*11819*	*91392*	*1356*	*1605*	*1346*	*1353*	*1174*	*1272*	*8106*
Ever had sex	*6542*	*7160*	*6840*	*6049*	*5645*	*4880*	*4170*	*41286*	*438*	*558*	*487*	*423*	*334*	*358*	*2598*
Sexually active‡	*4849*	*5277*	*4890*	*4430*	*4088*	*3519*	*3029*	*30082*	*276*	*346*	*300*	*242*	*200*	*234*	*1598*
**Distribution of respondents**	* *							* *							* *
**Sex (%)**															
Female	49.0	47.4	47.9	49.9	48.1	50.3	49.1	48.8	45.9	47.3	47.8	46.1	47.6	49.3	47.3
Male	51.0	52.6	52.1	50.1	51.9	49.7	50.9	51.2	54.1	52.7	52.2	53.9	52.4	50.7	52.7
**Race/ethnicity (%)**															
White^§^	60.8	59.1	57.4	56.6	55.2	54.2	51.8	56.5	55.8	54.9	49.1	53.3	52.6	50.4	52.8
Black^§^	15.1	14.5	14.1	14.2	13.4	13.1	12.2	13.8	15.1	16.1	16.1	13.8	13.6	13.9	14.8
Hispanic/Latino	16.4	18.2	19.7	20.7	22.0	22.5	25.6	20.7	17.4	19.3	22.9	23.0	23.0	25.6	21.7
All Other Races	7.7	8.2	8.7	8.5	9.4	10.1	10.4	9.0	11.7	9.7	11.9	10.0	10.8	10.1	10.7
**Age (%)**															
15	29.5	28.0	28.1	26.9	29.1	28.4	28.2	28.3	32.3	30.0	26.8	30.3	27.0	28.2	29.2
16	29.2	29.3	29.6	28.2	28.0	28.9	29.2	28.9	29.3	26.4	32.4	27.9	31.4	29.4	29.4
17	26.3	27.4	27.1	27.5	26.4	27.5	27.0	27.0	26.2	29.8	28.3	30.6	26.8	31.8	28.9
18–19‖	15.1	15.3	15.2	17.4	16.5	15.2	15.6	15.7	12.2	13.9	12.5	11.3	14.8	10.6	12.5

* Analytic sample contains respondents in high school (ages 15–18+).

† The years displayed are midpoints of data releases 2006–08, 2008–10, 2011–13, 2013–15, 2015–17, and 2017–19.

‡ For this analysis, contraceptive use is limited to respondents who have had sex in past three months.

§ NSFG specifies non-Hispanic White and Black.

‖ The YRBS only indicates 18 years or older.

**Fig 1:**
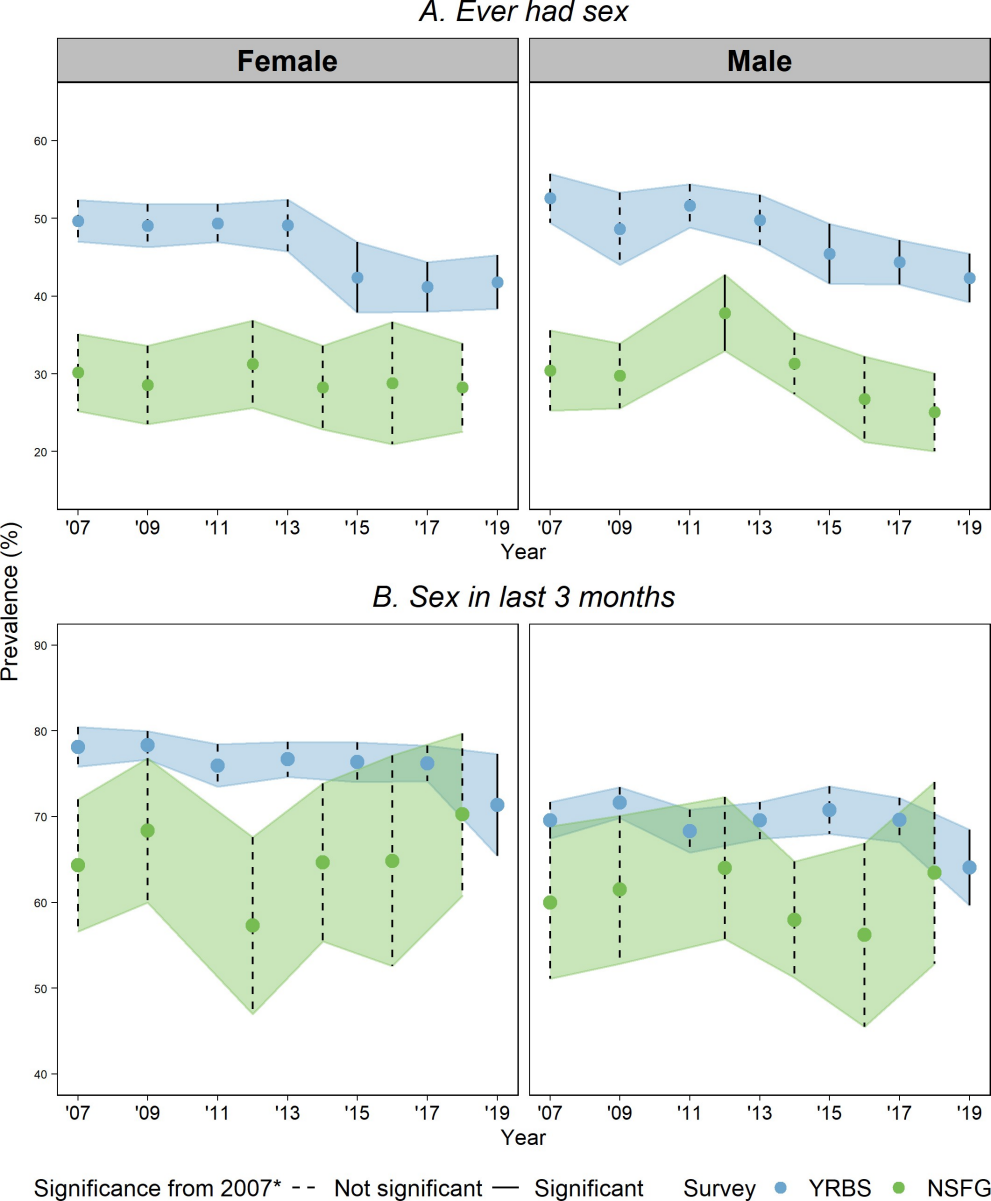
Prevalence of ever had sex and sex in last 3 months by sex, NSFG and YRBS, 2007–2019. (A) Ever had sex (B) Sex in last 3 months; Prevalence is plotted for YRBS (blue) and NSFG (green) with 95% confidence intervals (CIs). Significant difference from prevalence in 2007 displayed as solid CI and not-significant as dotted. * p < 0.05, Note: The years displayed for the NSFG are midpoints of data releases 2006–08, 2008–10, 2011–13, 2013–15, 2015–17, and 2017–19.

**Fig 2:**
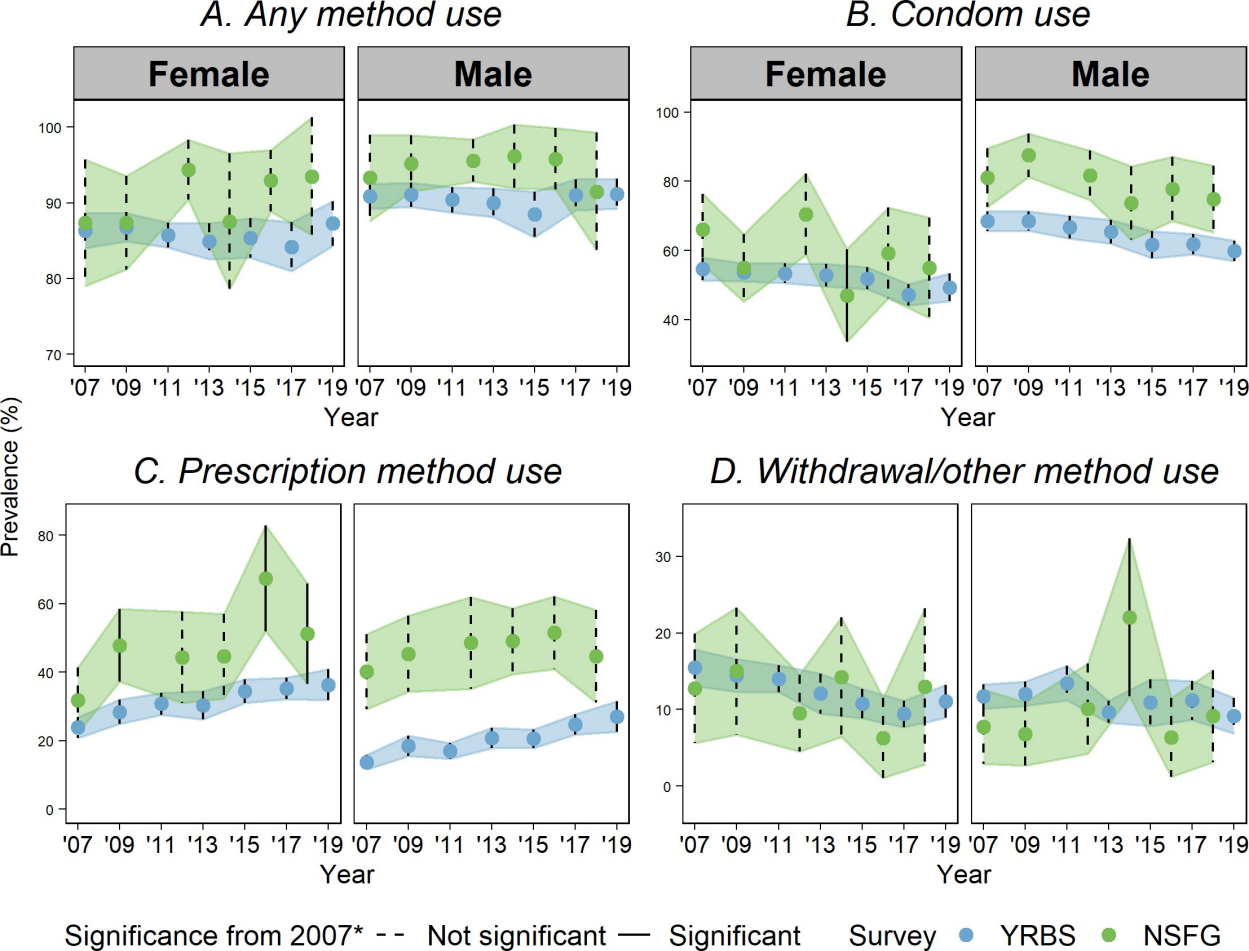
Prevalence of contraceptive method type at last sex by sex, NSFG and YRBS, 2007–2019. (A) Any method use (B) Condom use (C) Prescription method use (D) Withdrawal/other method use; Prevalence is plotted for YRBS (blue) and NSFG (green) with 95% confidence intervals (CIs). Significant difference from prevalence in 2007 displayed as solid CI and not-significant as dotted. * p < 0.05, Note: The years displayed for the NSFG are midpoints of data releases 2006–08, 2008–10, 2011–13, 2013–15, 2015–17, and 2017–19.

Figs 1 and 2 graph the estimated prevalence and 95% CI for each outcome over time separately by survey (full regression results available in S2–S7 Tables). Any year that has a statistically different point estimate (p < .05) from 2007 is indicated in the figures with a solid CI bar. Findings for each variable are presented below.

**Ever had sex:** The proportion of in-school adolescents who ever had sex was lower in the NSFG than the YRBS consistently over time for both sexes, with non-overlapping confidence intervals between paired or adjacent years. For example, by the end of the observed timeframe, the estimated proportion of females who ever had sex in the 2019 YRBS and the 2018 NSFG was 42% (CI 38–45) and 28% (CI 23–34), respectively. Here, and for each of the other outcomes, the confidence intervals are larger for the NSFG than the YRBS, given the NSFG’s smaller sample size.

The YRBS documents significant declines from 2007 to 2019 in the proportion of sexually experienced students for both male and female students. In contrast, the NSFG generally showed no significant changes over time in the prevalence of sexual experience for either males or females; the one exception was a significant increase among males from 2007 to 2012, which was not sustained. The YRBS had a similar uptick; although not significant, it was an interruption in the clear downward trend over the longer period under study.

**Current sexual activity:** The proportion of currently sexually active adolescents, among those who ever had sex, was generally larger in the YRBS than the NSFG in comparable periods, although with small overlaps in confidence intervals for some years. In the YRBS, this predicted probability declined significantly from 2017 to 2019 for both males and female students. There was no other significant change over time in either survey.

**Any contraceptive use at last sex:** The proportion of sexually active respondents reporting any contraceptive use at last sex was statistically indistinguishable between the two surveys at most time points. However, the point estimates were consistently higher in the NSFG. Neither survey system had significant changes over time.

**Any prescription method use at last sex:** The prevalence of prescription method use at last sex among sexually active females and males was substantially higher in the NSFG than the YRBS with primarily non-overlapping CIs over time (all but the most recent CIs for men in the NSFG are non-overlapping with the YRBS, and three CIs for women). The absolute differences in estimated prevalence between surveys got larger towards the end of the observed period.

Within the YRBS, we saw significant increases in the prevalence of use for males and females. Within the NSFG, there was no significant change among males (although the generally upward direction of estimates paralleled the YRBS). Among females, there were significant increases compared to 2007 in all but the 2012 and 2014 estimates.

**Any condom use at last sex:** Throughout the period, more males reported condom use at last sex in the NSFG than the YRBS, with generally non-overlapping CIs (only the 2014 NSFG overlapped with contemporaneous YRBS estimates). Among females, the NSFG point estimates for condom use were higher than the YRBS, but the CIs were all overlapping. Compared to 2007, the YRBS estimates significant declines in condom use among females in 2017 and 2019, while the NSFG estimates a significant decline only in 2014. Among males, condom use declined significantly only in recent years of the YRBS, although the direction of change was similar in the NSFG.

**Any withdrawal use at last sex:** For both sexes, the proportion of sexually active respondents reporting use of withdrawal at last sex was statistically indistinguishable between the two surveys at most time points. Among females, prevalence declined significantly in the YRBS but not the NSFG. In both surveys, estimates remained stable among males, except for a not sustained large increase from in 2014 in the NSFG.

### Sensitivity analyses

**Fig 3:**
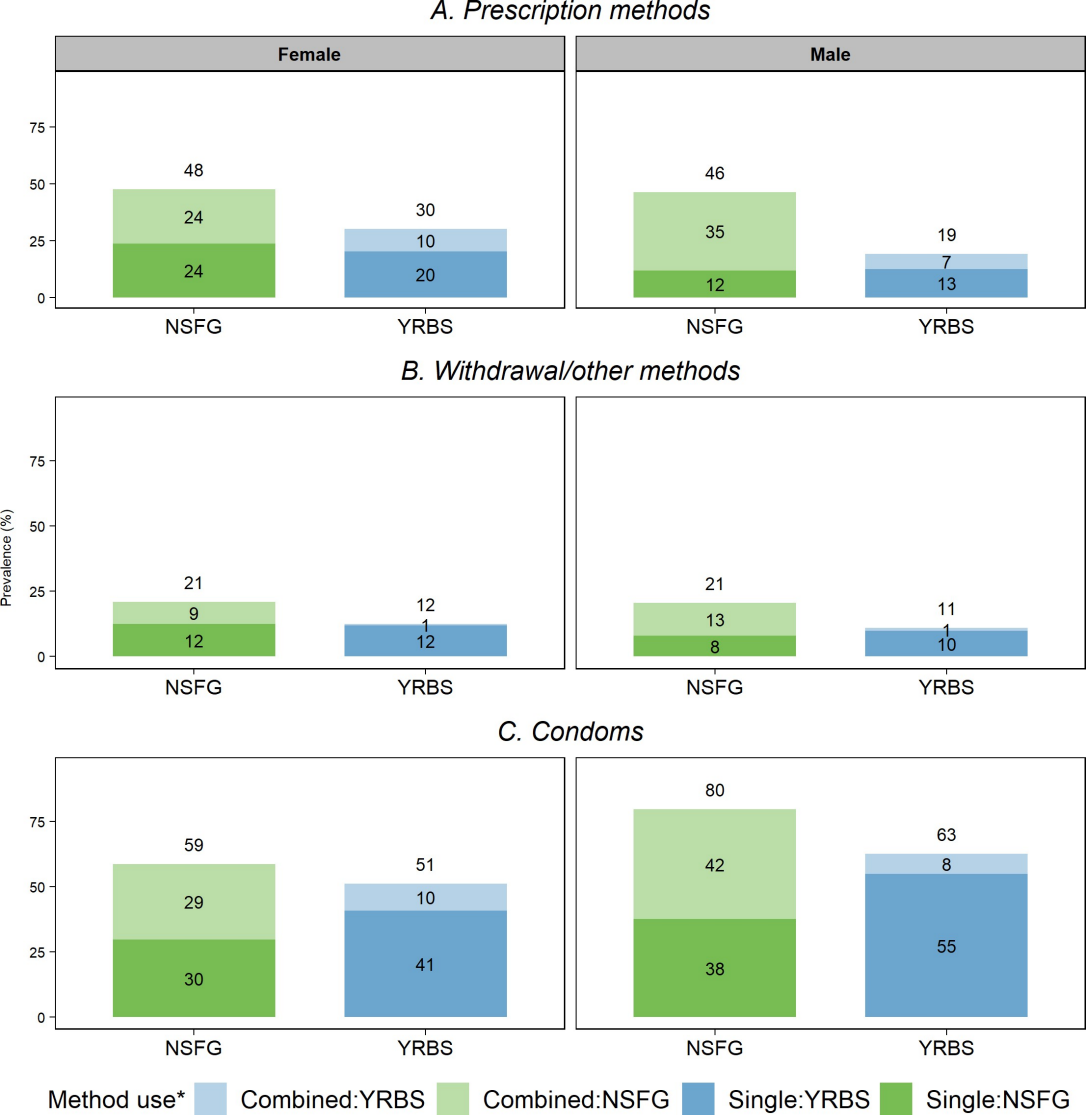
Overall, combined, and single use of selected contraceptive methods at last sex by survey and sex, 2007–2019. (A) Prescription methods (B) Withdrawal/other methods (C) Condoms; * Combined use for prescription and withdrawal methods included respondents who reported using these methods and condoms. Combined use for condoms included respondents using this method and either prescription methods or withdrawal.

After pooling the data from 2007 to 2019, we found that the overall prevalence of withdrawal or prescription method use alone was comparable between the two surveys (Fig 3). However, the prevalence of condom use with these methods was markedly higher in the NSFG than the YRBS for both males and females. For example, in the NSFG, 24% of females using prescription methods and 9% using withdrawal reported also using condoms; these proportions dropped to 10% and 1%, respectively, in the YRBS. Furthermore, in the NSFG 29% of females reported condom use with another method, compared to 10% in the YRBS. These survey patterns are similar among males as well.

Second, we estimated greater differences between surveys in the reported share of respondents ever having sex at younger ages (S8 Table). Among males, the YRBS shows significantly higher likelihood of ever having sex among 15-year-olds than in the NSFG (odds ratio 3.1, CI 2.4–3.8) and smaller but still significant differences among 18-19-year-olds (odds ratio 1.4, CI 1.1–1.8). Similar patterns were observed among females.

Using the NSFG sexual experience measures from the ACASI instead of the FTF interview did not substantially narrow the gap in estimates between the two survey systems (*results not shown*). Furthermore, fewer than 5% of YRBS respondents reported both ever having sex and only having same-sex partners (and thus not penile-vaginal intercourse). Removing this small group of YRBS respondents from the overall prevalence for the sexual activity measures did not close the gap between the two surveys’ prevalence estimates for these measures.

Finally, we found that the prevalence of recent alcohol use was significantly greater in the YRBS than the NSFG, paralleling the greater reporting of sexual risk behaviors in the former survey. In contrast, respondents’ reports of their height and weight, presumably less sensitive measures than alcohol use or sexual behaviors, did not vary significantly between surveys *(results not shown)*.

## Discussion

Our analyses of the YRBS and NSFG document inconsistent prevalence estimates and trends for the sexual behaviors under study during a recent period across comparable samples of in-school adolescents. Among this sample, the share reporting being sexually experienced was greater in the YRBS than in the NSFG. Although there was no evidence of significant differences between surveys in overall contraceptive use, patterns across specific methods differed and the NSFG showed greater use of more effective prescription methods than the YRBS. Combined with higher rates of sexual experience measured in the YRBS, the YRBS estimates more young people exposed to the risk of pregnancy and STIs than the NSFG.

The YRBS estimated significant declines over time in sexual experience, and recent declines in sex in the last three months, for both sexes, while the NSFG showed no change. Trends in contraceptive use were generally similar across surveys for females, while only the YRBS estimated significant changes among males. Differences between surveys in the prevalence of specific contraceptive methods reflect greater combined use of methods in the NSFG than the YRBS. Indeed, the surveys are not markedly different in their estimation of each method alone, or in overall contraceptive method use. The lower reporting of condom use combined with other methods in the YRBS compared to the NSFG likely reflects differences in the question wording and format between surveys. Method use in the NSFG is reported in a single question, and respondents can report multiple types of contraception used. In contrast, the measurement of combined method use in the YRBS requires analysts to combine responses to two separate questions, which elicits less dual method reporting than the single NSFG question. Additionally, the YRBS method use question only allows respondents to select one response, thus dual use with a method other than a condom cannot be identified. Further research is needed to understand how respondents interpret the two YRBS questions and how this influences reporting patterns.

Adolescents may underreport sensitive behaviors in household surveys out of concerns about privacy and confidentiality [22–27]. Prior research has interpreted that the higher prevalence of sexual experience reported in the YRBS reflects the greater privacy afforded by a self-administered classroom survey [6, 26]. However, this study found that the more confidential ACASI in the NSFG did not alleviate differences in reporting with the YRBS.

Instead, we believe that that these findings suggest that the YRBS likely overestimates rates of sexual experience, especially among younger respondents. First, the YRBS does not provide a precise definition for sexual intercourse; as a result, YRBS respondents may report sexual behaviors other than penile-vaginal intercourse, such as oral or anal sex. An unpublished 2000 study from the CDC showed that 93% of students defined vaginal sex as sexual intercourse, 62% of students defined anal sex as sexual intercourse, and 22% of students defined oral sex as sexual intercourse [28]. These different interpretations could be impacting the between-survey differences in the intercourse measures overall. Further evidence of differential interpretation comes from the patterns by age. In the NSFG, the share of young people engaging in oral sex but not penile-vaginal intercourse declines with age [29]. At older ages, when adolescents become more likely to engage in penile-vaginal intercourse, the differences between surveys in the prevalence of sexual experience narrows. If YRBS respondents are reporting on a broader definition of sex, this may also explain the lower reporting of contraceptive use among those self-identified as sexually active [1, 30]. Second, the role of social desirability bias in reporting in these data must be given greater consideration, particularly in the YRBS [31]. For example, estimated declines in sexual experience in the YRBS may be influenced by changing patterns of social desirability for this behavior. Additionally, being an in-school, self-administered survey, the YRBS may be more susceptible than the NSFG to motivated misreporting of sensitive behaviors, whether because of social desirability or other types of peer influence [32–34]. Indeed, alcohol use—another sensitive behavior for adolescents—also has a higher prevalence in the YRBS than the NSFG. Still, there may be other differences between the surveys that influence the reporting patterns estimated here.

We found more behaviors with statistically significant changes over time in the YRBS than the NSFG. Differences in sample size, and thus analytic power, may explain some of this. For example, although the NSFG sample used here was large enough to identify the same magnitude of changes in the prevalence of ever had sex as the YRBS, it was inadequately powered to identify the same changes in contraceptive use. The adolescent sample from the NSFG used in this analysis includes only about two-thirds of the full 15-19-year-old NSFG sample and thus other NSFG analyses of adolescents will have larger sample sizes than examined here.

### Limitations

Each survey had different designs and data collection techniques that could not be fully isolated in this analysis. Although the surveys covered similar periods, exact survey years and time intervals were not consistent. We could not identify the exact age of YRBS respondents 18 years or older, although only 3% of the full sample of NSFG respondents were attending high school and older than age 19. This suggests that nearly all respondents 18 years and older in the YRBS are 18 or 19 and thus comparable to the NSFG analytic sample. By narrowing the sample size of both surveys to create a common analytic sample, this reduced statistical power and the ability to examine population subgroups.

Although this study was not able to determine the specific underlying causes of differences between surveys, it provides a baseline descriptive analysis that can guide future research attempting to pinpoint causes. More research is needed to understand the impact of various survey design components, including interview length, data collection privacy and mode, question wording, and placement.

### Recommendations

Moving forward, we suggest adopting more explicit wording for the YRBS measures of sexual behavior, echoing recommendations from the recent National Academy of Sciences report [1]. We cannot assume that all young people share a common definition of sex, and the survey must provide more explicit guidance on this question. We also suggest making changes to the YRBS condom and contraception measures to obtain more complete reporting of dual use of condoms and other methods. At the same time, changing question wording in the YRBS is a lengthy process, which involves engagement with both subject matter experts and YRBS coordinators at all sites [35], and would have implications for consistency of tracking measures over time. Yet, efforts to update wording in the YRBS or NSFG are critical to improve the reliability of both survey systems. The CDC continues to strengthen the YRBS and the 2019 survey incorporated a number of positive changes [36]. As the 30-year anniversary for the YRBS approaches in 2021, we need to ensure that the data are accurately reflecting the experiences of today’s youth.

We also recommend increasing the NSFG sample size of adolescents. One of the NSFG’s purposes includes monitoring trends, but at present, it is underpowered to detect trends in some measures of sexual behavior among young people, particularly by demographic groups. However, the NSFG offers a far more robust set of individual and household level measures useful for multivariate analyses than the YRBS and offers many measures for understanding the circumstances of adolescent sexual and contraceptive behaviors, such as the voluntary status of sexual activity, partner age and other characteristics. As such, it is often relied upon for more comprehensive study of young people’s sexual and reproductive health. Improving the NSFG sample size of 15-19-year-olds could enable more meaningful overall and subgroup analyses, increasing the usefulness of these data for policymaking in adolescent populations.

Until larger system-level changes are made, pooling years of the NSFG data may provide opportunities for more robust research, especially for analyses on smaller populations such as sexually active adolescents or adolescents of color. The ability to do this is an advantage of the NSFG being a continuous survey with comparable measures that should be utilized. While it is common to pool the NSFG into four-year periods for analysis, even longer intervals may be adequate for some research questions. Even in the YRBS, pooling waves of data can allow for research on smaller subgroups [37].

### Implications

This study shows that public health surveillance may reach different conclusions about the patterns of adolescent sexual behaviors, depending on whether the YRBS or NSFG is examined. Without reliable external estimates, we cannot reach a precise conclusion about which surveys’ estimates are more valid. Users of these data will not find a one-size-fits-all approach to measurement concerns but should consider issues of social desirability and other reporting biases in the data and acknowledge limits in their research. This work highlights the need for caution when using specific data points to support public health policy decisions and actions. By design, each survey has a distinct mission and sample population that can provide relevant and targeted information about different groups and settings (i.e., school vs. community-based populations). The YRBS may be the more appropriate data source for studies that focus on school-based policies as practices, but the lack of generalizability to the full population of young people must be acknowledged. While the YRBS is a valuable resource on the behaviors of high school students, it does not provide information on out-of-school adolescents. As a result, data from the YRBS cannot inform policies for our most vulnerable young people—those less connected to systems of education, health care, services, and support. Data users, including researchers, policymakers, and the media, should carefully draw inferences from either survey, bearing in mind their samples, study designs, and limitations. Further efforts to improve these national data collection efforts can inform policy and research efforts that support adolescent sexual and reproductive health and wellbeing.

## Supporting information

S1 Table Variable definitions and question text for YRBS and NSFG with corresponding variable name in survey. (XLSX)

S2 Table Unadjusted logistic regression results of ever had sex, by year and survey, for females and males, and estimated prevalence* with 95% confidence intervals. (XLSX)

S3 Table Unadjusted logistic regression results of sex in last three months*, by year and survey, for females and males, and estimated prevalence† with 95% confidence intervals. (XLSX)

S4 Table Unadjusted logistic regression results of any contraceptive method use*, by year and survey, for females and males, and estimated prevalence† with 95% confidence intervals. (XLSX)

S5 Table Unadjusted logistic regression results of any prescription method use*, by year and survey, for females and males, and estimated prevalence† with 95% confidence intervals. (XLSX)

S6 Table Unadjusted logistic regression results of any condom use*, by year and survey, for females and males, and estimated prevalence† with 95% confidence intervals. (XLSX)

S7 Table Unadjusted logistic regression results of withdrawal use*, by year and survey, for females and males, and estimated prevalence† with 95% confidence intervals. (XLSX)

S8 Table Sexual behavior variables stratified by survey and age*, YRBS and NSFG: 2007–2019. (XLSX)
